# Neonatal systemic inflammation in rats alters retinal vessel development and simulates pathologic features of retinopathy of prematurity

**DOI:** 10.1186/1742-2094-11-87

**Published:** 2014-05-15

**Authors:** Hye Kyoung Hong, Hyun Ju Lee, Jung Hwa Ko, Ji Hyun Park, Ji Yeon Park, Chang Won Choi, Chang-Hwan Yoon, Seong Joon Ahn, Kyu Hyung Park, Se Joon Woo, Joo Youn Oh

**Affiliations:** 1 Department of Ophthalmology, Seoul National University Bundang Hospital, 300 Gumi-dong, Seongnam 463-707, Korea; 2 Laboratory of Ocular Regenerative Medicine and Immunology, Biomedical Research Institute, Seoul National University Hospital, 101 Daehak-ro, Jongno-gu, Seoul 110-744, Korea; 3 Department of Pediatrics, Seoul National University College of Medicine, Seoul National University Bundang Hospital, 300 Gumi-dong, Seongnam 463-707, Korea; 4 Department of Internal medicine, Cardiovascular Center, Seoul National University Bundang Hospital, 300 Gumi-dong, Seongnam 463-707, Korea; 5 Department of Ophthalmology, Seoul National University Hospital, 101 Daehak-ro, Jongno-gu, Seoul 110-744, Korea; 6 Department of Ophthalmology, Seoul National University College of Medicine, 101 Daehak-ro, Jongno-gu, Seoul 110-744, Korea

**Keywords:** angiogenesis, inflammation, lipopolysaccharide, retina, retinopathy of prematurity

## Abstract

**Background:**

Alteration of retinal angiogenesis during development leads to retinopathy of prematurity (ROP) in preterm infants, which is a leading cause of visual impairment in children. A number of clinical studies have reported higher rates of ROP in infants who had perinatal infections or inflammation, suggesting that exposure of the developing retina to inflammation may disturb retinal vessel development. Thus, we investigated the effects of systemic inflammation on retinal vessel development and retinal inflammation in neonatal rats.

**Methods:**

To induce systemic inflammation, we intraperitoneally injected 100 μl lipopolysaccharide (LPS, 0.25 mg/ml) or the same volume of normal saline in rat pups on postnatal days 1, 3, and 5. The retinas were extracted on postnatal days 7 and 14, and subjected to assays for retinal vessels, inflammatory cells and molecules, and apoptosis.

**Results:**

We found that intraperitoneal injection of LPS impaired retinal vessel development by decreasing vessel extension, reducing capillary density, and inducing localized overgrowth of abnormal retinal vessels and dilated peripheral vascular ridge, all of which are characteristic findings of ROP. Also, a large number of CD11c^+^ inflammatory cells and astrocytes were localized in the lesion of abnormal vessels. Further analysis revealed that the number of major histocompatibility complex (MHC) class II^lo^CD68^lo^CD11b^lo^CD11c^hi^ cells in the retina was higher in LPS-treated rats compared to controls. Similarly, the levels of TNF-α, IL-1β, and IL-12a were increased in LPS-treated retina. Also, apoptosis was increased in the inner retinal layer where retinal vessels are located.

**Conclusions:**

Our data demonstrate that systemic LPS-induced inflammation elicits retinal inflammation and impairs retinal angiogenesis in neonatal rats, implicating perinatal inflammation in the pathogenesis of ROP.

## Background

Normal development of retinal blood vessels is critical for vision. Aberration in retinal angiogenesis during development in preterm infants is a major cause of blindness in retinopathy of prematurity (ROP) that afflicts millions of the US population [1-3]. More than 80% of premature infants weighing less than 1,000 g develop ROP, and the incidence of ROP is rapidly increasing because of improved survival of the preterm infants [1-3].

Current therapeutic strategies for ROP include a tight control of environmental oxygen and laser retinal photocoagulation [4]. These treatments have been used based on the evidence that changing levels of oxygen and subsequent oxidative stress contribute to the pathogenesis of ROP [5]. However, the effect of low oxygen therapy on the long-term developmental outcome is not clear. A recent report showed that a reduction in occurrence of ROP by low oxygen therapy co-occurred with an increase in mortality [6], while some reports found no association between target oxygen levels and death rates [7]. Hence, the best oxygen level to reduce ROP has not been determined, and actual intervention by oxygen may not provide the optimal means to manage ROP [8]. Also, laser photocoagulation ablates peripheral avascular retina, and has unfavorable sequelae on visual acuity and field [9]. Therefore, efforts are being made to develop novel preventive and therapeutic modalities for ROP by targeting the biological pathway of ROP.

Although exposure of the immature retina to excessive oxygen is an important factor in ROP pathogenesis [5], there is an accumulating body of evidence that perinatal infection and inflammation are largely associated with an increased risk for ROP [10]. Epidemiological studies have shown that the incidence of ROP was higher in patients with early or late onset neonatal sepsis [10-14]. Also, some reports indicated a relationship between the development of ROP and the levels of pro-inflammatory cytokines such as TNF-α [15]. Other reports showed that infants born to mothers with chorioamnionitis or leukocytosis were at increased risk of ROP [16,17]. In addition to ROP, perinatal infection or inflammation has recently been associated with various developmental diseases of preterm infants such as bronchopulmonary dysplasia, necrotizing enterocolitis, periventricular leukomalacia, and cerebral palsy [18-20].

Based on the evidence, we hypothesized that systemic inflammation prior to completion of retinal development might disturb normal retinal angiogenesis, and subsequently lead to the development of ROP. To test this hypothesis, we here investigated the effects of systemic lipopolysaccharide (LPS) administration on vessel development, inflammatory cell and molecule expression, and apoptosis in the retina of neonatal rats.

## Methods

### Animals and animal model

The experimental protocols were approved by the Institutional Animal Care and Use Committee of Bundang Seoul National University Hospital Biomedical Research Institute (IACUC No. BA1302-123/011-01). Animals were treated in strict accordance with the ARVO statement for the use of animals in ophthalmic and vision research [21].

Pregnant Sprague–Dawley rats weighing 300 to 360 g were purchased from Orient Bio Inc. (Seongnam, Korea), and maintained in a specific pathogen-free environment with continuously available water and food. The female-to-male ratio ranged from 0.8 to 1.2, and sex differences between the groups were not statistically significant. The litters of the rats received intraperitoneal (IP) injections of 100 μl LPS (0.25 mg/kg; lot No. 2274257, Sigma-Aldrich, St. Louis, MO, USA) three times on days 1 (P1), 3 (P3), and 5 (P5) after birth. The same volume of normal saline (NS), which was LPS-free, was injected in the same manner in the control group. We used LPS because LPS is a well-known ligand for toll-like receptor(TLR) 4 that is involved in both infectious and sterile inflammation [22]. We used neonatal rats because retinal vessels develop during the first few weeks after birth in rodents mimicking the environment where ROP occurs in preterm human infants [23,24]. Rat pups were maintained in normoxia, and checked daily for body weight. There were no statistically significant differences in body weight between the LPS-treated rats and NS-treated controls. On P7 and P14, rat pups were anesthetized by an IP injection of ketamine (50 mg/kg; Yuhan, Seoul, Korea) and xylazine (50 mg/kg; Bayer AG, Leverkusen, Germany), and eyes were collected for analysis. One eye was subjected to histological assays, and the fellow eye was processed for flow cytometry or RT-PCR analysis.

### Histopathology

The eyes were enucleated and fixed in 2% paraformaldehyde/PBS (phosphate buffered solution, pH 7.4) for 5 min. The retinas were then isolated from eyeballs, and permeabilized with 0.5% Triton X-100, 5% FBS, and 20% DMSO in PBS for 3 h at room temperature (RT). For vessel staining, the retinas were incubated with BS-1 Lectin-FITC (Sigma-Aldrich), chicken anti- GFAP (Millipore, Temecula, CA, USA), rabbit anti- NG2 (Millipore) at 4°C for 4 days. The secondary antibodies used were Cy3-conjugated anti-chicken IgG and Alexa Fluor 633-conjugated anti-rabbit IgG (Molecular probes™, Carlsbad, CA, USA). For macrophage staining, the retinas were incubated with anti-CD11c-FITC (Serotec, Oxford, UK), CD11b (Abcam, Cambridge, UK), and CD68 (Bioss Antibodies, Woburn, MA, USA) at 4°C overnight. The secondary antibodies were Cy3-conjugated anti-rabbit IgG plus BS-Lectin-TRITC (Sigma-Aldrich). After staining, four cuts were made from the edges to the center of the retina, which was flattened and mounted with the vitreous side up on glass slides and visualized on a confocal microscope (LSM710, Carl Zeiss, Oberkochen, Germany).

For *in situ* apoptosis assay, the eyeballs were fixed in 4% paraformaldehyde for one day at RT, embedded in paraffin, and cut into serial 4-μm thick sections. The cross-sections were de-paraffinized using xylene and rehydrated in ethanol. The sections were permeabilized with 20 μg/ml Proteinase K (Gibco BRL, Carlsbad, CA, USA) in 10 mM Tris 7.5 and 5 mM EDTA for 15 min at RT. Then the slides were stained with TUNEL (terminal deoxynucleotidyl transferase dUTP nick end labeling) as the manufacturer’s protocol (Roche Diagnostics GmbH, Mannheim, Germany). Briefly, the slides were incubated in 50 μl of TUNEL cocktail in humidified chamber for 60 min at 37°C in the dark. Then, the slides were rinsed with PBS, and incubated with converter-POD (for anti-fluorescein antibody Fab fragment conjugated with horse-radish peroxidase) for 30 min at 37°C. After substrate reaction with DAB, the stained slides were visualized under light microscope.

In addition, the thickness ratio of the inner retina (retinal nerve fiber layer, ganglion cell layer, and inner plexiform layer) to the total retina was measured in cross-section images 1 mm from the optic nerve head.

### Vessel scoring and analysis

Images were obtained under a confocal laser scanning microscope (LSM710), and processed and analyzed using image analysis software (Zen 2011, Carl Zeiss, Oberkochen, Germany and Image J, NIH) at 100× magnification as follows. To score the vessel development, four to six non-overlapping fields (1 mm [2], 1 to 1.5 mm distance from optic nerve) were randomly selected per retinal flap in each flat mount. The vessel extension was measured as the length (μm) of the vessel from the optic nerve to the border between vascularized and nonvascularized retina. The number of branch points and vessel net holes was measured in the unit area (mm [2]) of peripheral retina. The vessel density was determined using a 1 × 1 grid (grid element side length approximately 1,000 μm) in the confocal images (magnification, 100×).

### Real-time reverse transcriptase PCR

For RNA extraction, retinas were minced into small pieces, lysed in RNA isolation reagent (RNA Bee; Tel-Test, Friendswood, TX, USA), and homogenized using a sonicator (Ultrasonic Processor, Cole Parmer Instruments, Vernon Hills, IL, USA). Total RNA was extracted using RNeasy Mini kit (Qiagen, Valencia, CA, USA) and used to synthesize double-stranded cDNA by reverse transcription (SuperScript III; Invitrogen, Carlsbad, CA, USA). Real-time amplification was performed using TaqMan Universal PCR Master Mix (Applied Biosystems, Carlsbad, CA, USA). For all the PCR probe sets, Taqman Gene Expression Assay kits were purchased from Applied Biosystems. The assays were performed in dual technical replicates for each biological sample.

### Flow cytometry

The retinas were placed and minced between the frosted ends of two glass slides in RPMI media (Welgen, Daegu, Korea) containing 10% fetal bovine serum and 1% penicillin-streptomycin. Cell suspensions were collected, and incubated for 30 min at 4°C with anti-MHC class II (ebioscience, San Diego, CA, USA), anti-CD11b (BD BioSciences, Mountain View, CA, USA), anti-CD11c (Serotec), and anti-CD68 anticodies (Serotec). The cells were assayed for fluorescence using a FACSCanto flow cytometer (BD BioSciences). Data were analyzed using Flowjo program (Tree Star, Inc., Ashland, OR, USA).

### Statistical analysis

The data are presented as the mean ± SEM. Comparisons of two values between the groups were made using the two-tailed Student’s *t* or Mann–Whitney test, and comparisons of more than two means were made using a one-way ANOVA (Prism™, GraphPad Software, Inc., La Jolla, CA, USA). Differences were considered significant at *P* <0.05.

## Results

### Postnatal systemic lipopolysaccharide administration impaired normal retinal vessel development

**Figure 1:**
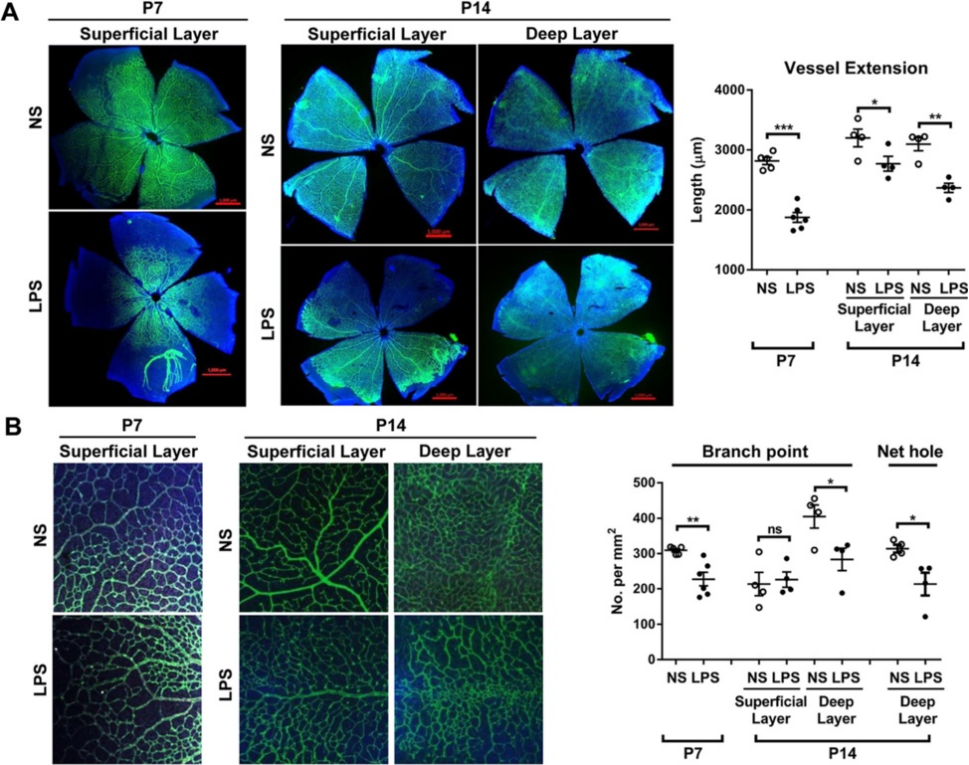
**Systemic lipopolysaccharide (LPS) altered retinal vessel development in neonatal rats. A**. The lectin staining of the retinal whole mounts from P7 and P14 rats showed that centrifugal growth of retinal vessels was significantly reduced by postnatal systemic LPS administration as measured by the length of vessels between the optic nerve and the peripheral vascular edge. Scale bars = 1000 μm. **B**. Retinal capillary density as measured by the number of branch points and net holes of capillaries was also reduced in LPS-treated rats. *P <0.05; **P <0.01; ***P <0.001; ns, not significant.

On P7 and P14, the rats were humanely killed and the retinas were assayed for retinal vessels. The lectin staining of the whole retinal flat mounts showed that retinal vascular growth was significantly delayed in the LPS-treated rats, compared to the NS-treated controls (Figure 1). On P7 when retinal vessels grow radially from the optic nerve to the ora serrata within the superficial layer of the retina [23,24], vascular extension was significantly reduced in the superficial layer as measured by the length of retinal vessels between the optic nerve and the peripheral edge of the vessels (Figure 1A). Similarly, capillary density in the superficial plexus was also decreased in the LPS-treated group as measured by the number of branch points of capillaries (Figure 1B). On P14 when the vessels start to sprout downward and form the deep vascular plexus [23,24], the radial extension of the vessel to the peripheral edge was significantly reduced in both superficial and deep layers in the LPS-treated rats (Figure 1A). Also, capillary density in the deep plexus was significantly lower in the LPS-treated retina as assayed by the number of branch points and net holes of capillaries (Figure 1B).

**Figure 2:**
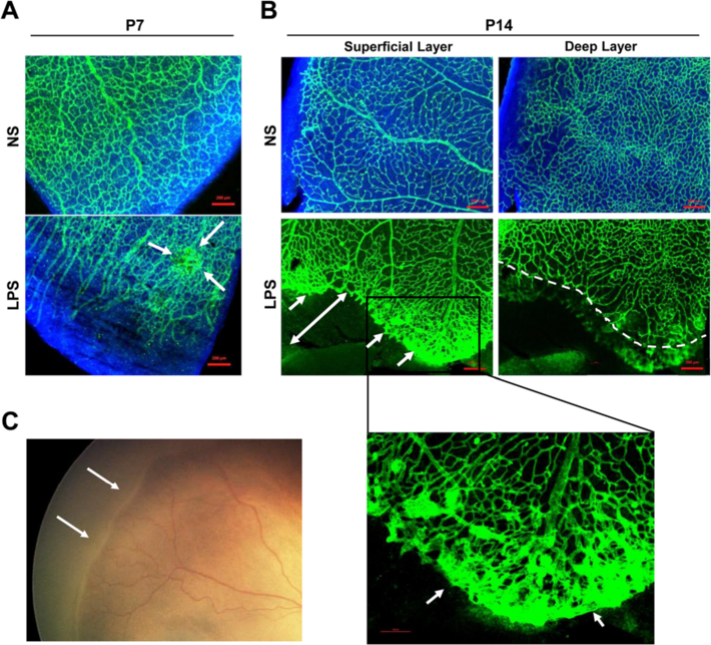
**Systemic lipopolysaccharide (LPS) induced retinopathy of prematurity (ROP)-like changes in peripheral margins of retinal vasculature. A**. On P7 retina in rats treated with LPS, the growth of peripheral retinal vessels was markedly delayed, and focal vascular tufts and abnormal angiogenesis developed (arrows). Scale bars = 200 μm. **B**. On P14 retina in the LPS-treated rats, the peripheral vascular margin in the superficial layer had abrupt vascular termination and tortuosity of surrounding vessels with a wide area of avascular retina (arrows) in contrast to controls. In the deep layer, a centrifugal growth of retinal vessels was markedly delayed (dashed line) compared to controls (arrows), and vascular density was also decreased. Scale bars = 200 μm. **C**. The ridge formation (arrows) and vascular tortuosity in the peripheral margin of vascularized retina are characteristic of retinopathy of prematurity in humans and similar to the findings of P14 retina in neonatal rats treated with systemic LPS **(B)**.

In addition to the delay in vessel growth and decrease in capillary density, abnormal vascular findings were also observed in the vascular edge of the peripheral retina (Figure 2). On P7, the peripheral retinal vessels in the LPS-treated group showed delayed centrifugal growth compared to the control, and had multiple focal vascular tufts (Figure 2A). The vascular dilatation and tortuosity were markedly increased in the peripheral retina of the LPS-treated group on P14, and a line of anastomosis developed at the peripheral edge where the vessels were abruptly terminated (Figure 2B). These findings in the peripheral retinal vessels are similar to the ridge formation and plus sign (vascular tortuosity and dilatation) that are commonly observed in stage 2 (pre-proliferative) ROP (Figure 2C).

### Systemic lipopolysaccharide induced CD11c^+^ cell infiltration and inflammatory cytokine expression in the retina

**Figure 3:**
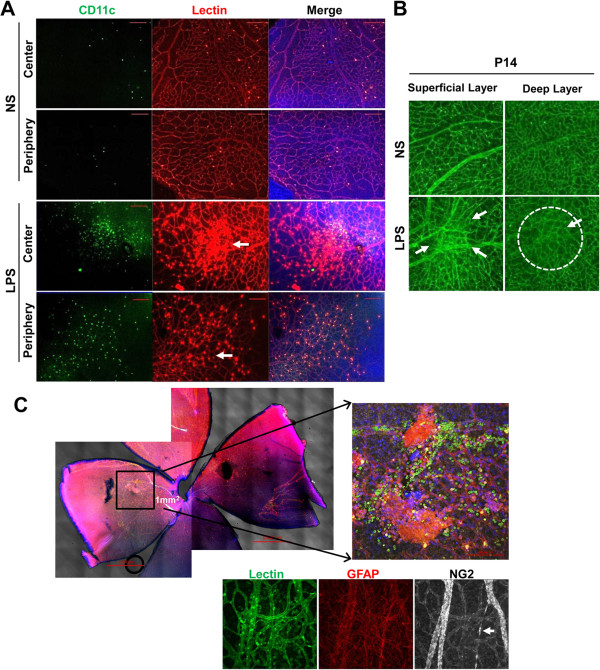
**Systemic lipopolysaccharide (LPS) recruited inflammatory cells in retina. A**. Immunohistochemical staining of retinal flat mounts from P7 rats showed a massive infiltration of CD11c^+^ cells around the area of focal abnormal vascular proliferation (arrows) in central and peripheral retina. Scale bars = 200 μm. **B**. On P14 retina, there were focal proliferations of abnormal retinal vessels in the superficial vascular layer (arrows). In the deep layer corresponding to the superficial layer of abnormal vascular proliferation, there were scanty capillaries indicating concomitant vascular regression (dashed circle). **C**. In the areas of focal vascular proliferations, infiltration of CD11c^+^ cells and proliferation of GFAP-stained astrocytes were noted. Also, regression of pre-existing capillaries was accompanied as indicated by ghost sheathing of NG2-stained pericytes (arrow). Scale bar = 1000 μm.

We next evaluated the inflammatory cells and molecules in the retina after systemic LPS administration. Immunohistochemical staining of retinal flat mounts showed that a large number of CD11^+^ cells infiltrated both central and peripheral retina of the LPS-treated rats on P7 (Figure 3A). Of note, the cells were largely localized in the area of abnormal retinal vessels. Neither CD68^+^ nor CD11b^+^ cells were observed (data not shown). Moreover, abnormal proliferation of astrocytes as well as CD11c^+^ cells infiltration was accompanied in the areas of focal vascular proliferation in the LPS-treated rats (Figure 3A). In the deep layer corresponding to the superficial retinal layer that had abnormal angiogenesis, capillary densities were decreased indicating concomitant vascular regression (Figure 3B). Regression of pre-existing capillaries was also observed (Arrow, Figure 3C).

**Figure 4:**
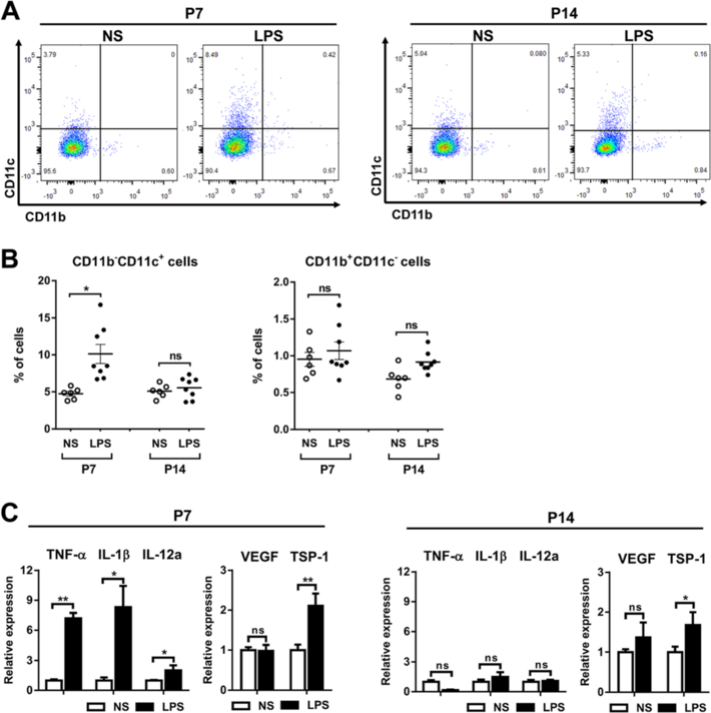
**Characteristics of inflammatory cells and molecules in retina after systemic lipopolysaccharide. A**, **B**. Flow cytometry revealed that the percentage of major histocompatibility complex (MHC) class II^lo^CD68^lo^CD11b^lo^CD11c^hi^ cells in the retina was increased in the LPS-treated group on P7, and normalized on P14. There was no difference in the percentage of CD11b^hi^ cells between the LPS- and normal saline (NS)-treated retinas. **C**. Real time RT-PCR showed that the levels of TNF-α, IL-1β, and IL-12a transcripts were increased in the retina of the LPS-treated group on P7, and returned to baseline on P14. Also, the level of thrombospondin (TSP)-1, an anti-angiogenic and pro-apoptotic factor, was significantly increased in the retina of the LPS-treated rats, while the level of VEGF transcript was not altered. *P <0.05; **P <0.01; ns, not significant.

Consistent with the immunohistochemical finding, flow cytometric analysis of retinal cells revealed that the percentage of MHC class II^lo^CD68^lo^CD11b^lo^CD11c^hi^ cells were significantly higher in the LPS-treated rats on P7 and normalized on P14 (Figure 4A). There was no difference in the percentage of CD11b^hi^ cells between the LPS- and NS-treated retinas (Figure 4A). The cells expressing CD68 were not detected among retinal cells.

We further analyzed the retina for inflammation- and angiogenesis-related cytokines. Real time RT-PCR assay revealed that the levels of TNF-α, IL-1β, and IL-12a transcripts in the retina were increased by systemic LPS on P7, and returned to normal levels on P14 (Figure 4B). Interestingly, the level of thrombospondin (TSP)-1, an anti-angiogenic and pro-apoptotic factor [25,26], was markedly increased in the retina of the LPS-treated rats on P7, while the level of VEGF transcript was not altered by LPS (Figure 4B).

### Systemic lipopolysaccharide induced apoptosis in the retina

**Figure 5:**
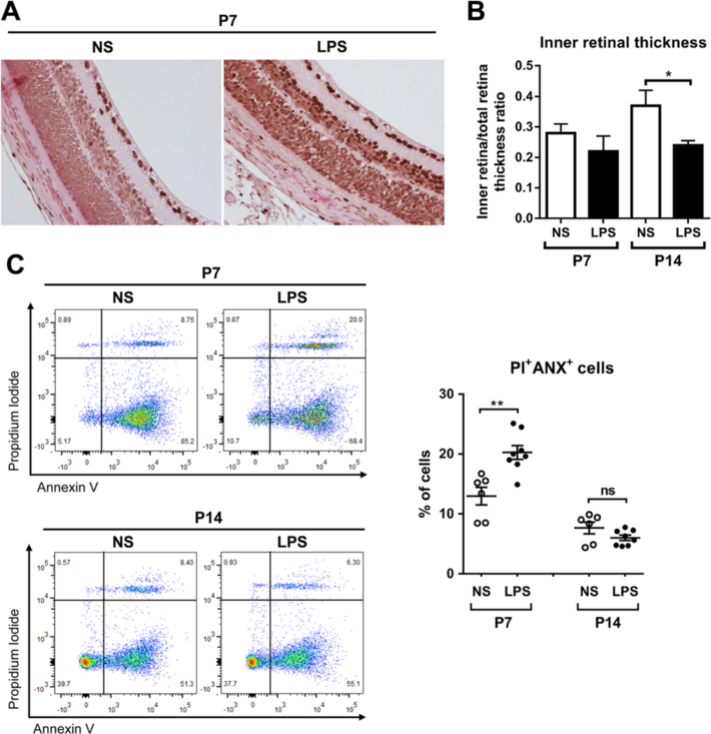
**Systemic lipopolysaccharide (LPS) induced apoptosis in retina. A**. TUNEL staining of retinal cross-sections from P7 rats indicated an increased number of apoptotic cells, most of which were located in the inner retinal layer, especially in the ganglion cell layer. Original magnification 400×. **B**. The thickness of inner retina relative to outer retina was significantly decreased in the LPS-treated P14 rats. **C**. Flow cytometry showed that the percentage of PI^+^Annexin^+^ cells indicating apoptotic cells were significantly increased in the retina of the LPS-treated P7 rats. *P <0.05; **P <0.01; ns, not significant.

Apoptosis of retinal cells including vascular endothelial cells is one of the causative factors in ROP development [27,28]. Therefore, we further assayed the retina for apoptosis. TUNEL staining of retinal cross-sections showed that a number of TUNEL-positive cells indicating injured or apoptotic cells were present in the inner retinal layer (where retinal vessels are located) of LPS-treated rats on P7, while there were fewer TUNEL-positive cells in the retina of NS-treated rats (Figure 5A). In addition, the thickness of the inner retinal layer was significantly decreased in the LPS-treated rats compared to the controls on P14 (Figure 5B). Flow cytometry showed that the proportion of PI^+^Annexin^+^ cells indicating apoptotic cells were significantly increased in the retina by LPS on P7 (Figure 5C). There was no difference in the number of apoptotic cells on P14 between LPS- and NS-treated groups (Figure 5C).

## Discussion

Data demonstrate that systemic LPS administration markedly altered retinal vessel development in neonatal rats by delaying vascular growth, reducing capillary density in the superficial and deep plexus, and forming aberrant vessel tufts in the peripheral retina. These findings are similar to vascular features of ROP [28]. Of note, abnormal retinal vessel development was accompanied by inflammatory cell infiltration, increased level of pro-inflammatory cytokines, and apoptosis. LPS is an endotoxin and TLR4 agonist that triggers inflammation [22]. Hence, our data suggest that systemic and retinal inflammation induced by LPS might result in dysregulation of vascular development and disrupt the balance between pro-angiogenesis and anti-angiogenesis in the retina.

The major risk factors for ROP are low gestational age, early exposure to high levels of oxygen, and late exposure to relatively lower oxygen levels [5,29]. Recently, there is an emerging body of clinical evidence that infants exposed to perinatal infection or inflammation are more susceptible to ROP [10]. In addition to clinical observations, Tremblay *et al*. [30] recently reported that perinatal LPS-induced inflammation without concurrent oxygen manipulation perturbed vascular development in the developing mouse retina. They observed an early increase in retinal vascular density and late depletion of retinal vascular beds in neonatal mice after systemic LPS administration. These are different from our findings of delayed centrifugal growth of retinal vessels and abnormal vascular proliferation. This difference might be related to the timing or severity of inflammation induced. Tremblay *et al*. [30] injected LPS once at P4, whereas we injected LPS three times at P1, P3, and P5. Thus, it is possible that the early and sustained inflammation in our model triggered more severe aberration in retinal vessel development similar to ROP. Also, Tremblay *et al*. [30] used mice for the study, and we used rats. The species difference might be another reason of different vascular features between the two studies. Nonetheless, these experimental data serve as another evidence to support the notion that systemic inflammation in the period when retinal development is not accomplished is one of the pathogenic mechanisms leading to ROP.

The mechanisms by which systemic inflammation is involved in retinal vessel development are not clear. One explanation is that systemic inflammation might indirectly affect retinal vessel growth by making immature retina vulnerable to hypoxic-ischemic injury. The rats in our study were maintained in normoxia and subject to neither hypoxic-ischemic injury nor variations in oxygen supplementation. Also, abnormal vascular proliferation within the vascular bed in our model did not accompany the capillary dropout (vasoattenuation) that is a characteristic finding of retinal hypoxia and typically observed in the conventional OIR model. Therefore, we speculate the vascular proliferation observed in our model might not be a result of retinal hypoxia. Nevertheless, we cannot rule out the possibility that systemic and retinal inflammation might cause retinal hypoxia, and thereby induce abnormal retinal angiogenesis. Another explanation is that inflammation might change the balance between pro-angiogenic and anti-angiogenic factors. Although there was no change in VEGF that is a main driver for angiogenesis, the level of TSP-1, an endogenous inhibitor of angiogenesis [31], was markedly increased in the retina by systemic LPS in our study. TSP-1 plays an important role in retinal angiogenesis during development. In mice overexpressing TSP-1, normal retinal vascular development was attenuated and vessel obliteration was increased [26]. The inhibitory effect of TSP-1 on retinal vessels was mediated by promoting apoptosis of vascular endothelial cells [32]. Also, TSP-1 was shown to antagonize VEGF-mediated signaling, and capillary survival in the developing retina was increased in mice lacking allele for TSP-1 [32]. Another report indicated that oxidative stress induced TSP-1, and thereby resulted in microvascular degeneration in a model of oxygen-induce retinopathy [25]. The relevance of TSP-1 in altered retinal vessel development in our model of systemic inflammation-induced retinopathy needs to be elucidated in further studies.

As for the mechanisms of how systemic inflammation induces retinal inflammation, many studies have been performed in the brain. Similar to ROP, intrauterine infection or neonatal sepsis is significantly associated with the development of cerebral palsy or neurodevelopmental disabilities which is termed ‘encephalopathy of prematurity’ [33-35]. Also, either maternal or neonatal systemic administration of LPS displayed brain damage in rat offspring [36,37]. When it comes to how TLR ligands in systemic circulation induce brain inflammation, some studies suggest that circulating LPS destructs the integrity of the blood–brain barrier by releasing cytokines from circulating inflammatory cells to induce brain inflammation [22,38-40]. Others suggest that systemic TLR ligands bind to brain endothelial cells and transmit inflammatory signals into the brain [22,41-43]. In our study, a large number of CD11c^+^ cells extravasated and infiltrated the lesion of abnormal retinal vessels, suggesting that activated inflammatory cells by systemic LPS might cross the blood-retinal barrier and affect normal retinal vascular growth in developing retina. Further study to identify these cells will help elucidate the mechanism of inflammation mediating abnormal vascular development in the retina of neonatal rats and eventually, ROP in humans.

There are several differences between our model and a well-known model of oxygen-induced retinopathy (OIR) [44]. First, retinal capillary obliteration and neovascularization were less prominent in our model, compared to the OIR model. This might be because retinal ischemia is less severe in the LPS-treated rats than in rats with OIR. Second, we observed a ridge formation in the peripheral vascular boundary in our model which is the essential step for severe ROP in humans. Hence, the retinal findings in our model are more similar to pathologic features of ROP in humans, compared to the conventional OIR model which manifests with severe capillary dropout and rarely shows peripheral ridge formation. Another feature of our model is abnormal vascular proliferation in the absence of hypoxia. This is in contrast to capillary dropout, a typical finding of the OIR model, and conflicts with the well-known mechanism of neovascularization in ROP: oxygen-induced capillary obliteration [40]. Additionally, vascular malformation in our model was accompanied by inflammatory cell infiltration. Thus, our data may add new knowledge to the mechanism of ROP.

## Conclusions

In conclusion, we here demonstrate that systemic LPS-induced inflammation elicits inflammation in the retina and altered retinal vessel growth similar to pathologic features of ROP. Our study indicates that modulation of inflammation may help elucidate the mechanism for retinal angiogenesis and provide the novel avenue of exploration for prevention and treatment of ROP.

## Abbreviations

CD: cluster of differentiation; IL: interleukin; IP: intraperitoneal; LPS: lipopolysaccharide; OR: oxygen-induced retinopathy; P: postnatal day; ROP: retinopathy of prematurity; RT: room temperature; TLR: toll-like receptor; TNF: tumor necrosis factor; TSP: thrombospondin; TUNEL: terminal deoxynucleotidyl transferase dUTP nick end labeling; VEGF: vascular endothelial growth factor.

## Competing interests

The authors declare that no competing financial or non-financial interests exist.

## Authors’ contributions

HKH carried out animal experiments and performed histological analyses. HJL carried out flow cytometric and molecular assays. JHK carried out flow cytometric and molecular assays. JHP helped to perform animal experiments and histological analyses. JYP helped to perform animal experiments and histological analyses. CWC participated in the design and coordination, and helped to draft the manuscript. CY helped with vessel staining. SJA helped with analysis of retinal vessels. KHP conceived of the study, participated in its design and coordination, and helped to draft the manuscript. SJW conceived of the study, participated in its design and coordination, analyzed the histological data, and helped to draft the manuscript. JYO conceived of the study, designed and coordinated the study, performed molecular assays, analyzed flow cytometric and molecular data, and wrote the draft. All authors read and approved the final manuscript.
